# The “Ram Effect”: A “Non-Classical” Mechanism for Inducing LH Surges in Sheep

**DOI:** 10.1371/journal.pone.0158530

**Published:** 2016-07-06

**Authors:** Claude Fabre-Nys, Audrey Chanvallon, Joëlle Dupont, Lionel Lardic, Didier Lomet, Stéphanie Martinet, Rex J. Scaramuzzi

**Affiliations:** 1 UMR 7247 Physiologie de la Reproduction et des Comportements, CNRS, INRA, Université de Tours, Institut français du cheval et de l’équitation, 37380 Nouzilly, France; 2 Department of Comparative Biomedical Sciences, Royal Veterinary College, Hawkshead Lane South Mimms, Hertfordshire AL9 7TA, United Kingdom; University of Florida, UNITED STATES

## Abstract

During spring sheep do not normally ovulate but exposure to a ram can induce ovulation. In some ewes an LH surge is induced immediately after exposure to a ram thus raising questions about the control of this precocious LH surge. Our first aim was to determine the plasma concentrations of oestradiol (E2) E2 in anoestrous ewes before and after the “ram effect” in ewes that had a “precocious” LH surge (starting within 6 hours), a “normal” surge (between 6 and 28h) and “late» surge (not detected by 56h). In another experiment we tested if a small increase in circulating E2 could induce an LH surge in anoestrus ewes. The concentration of E2 significantly was not different at the time of ram introduction among ewes with the three types of LH surge. “Precocious” LH surges were not preceded by a large increase in E2 unlike “normal” surges and small elevations of circulating E2 alone were unable to induce LH surges. These results show that the “precocious” LH surge was not the result of E2 positive feedback. Our second aim was to test if noradrenaline (NA) is involved in the LH response to the “ram effect”. Using double labelling for Fos and tyrosine hydroxylase (TH) we showed that exposure of anoestrous ewes to a ram induced a higher density of cells positive for both in the A1 nucleus and the Locus Coeruleus complex compared to unstimulated controls. Finally, the administration by retrodialysis into the preoptic area, of NA increased the proportion of ewes with an LH response to ram odor whereas treatment with the α1 antagonist Prazosin decreased the LH pulse frequency and amplitude induced by a sexually active ram. Collectively these results suggest that in anoestrous ewes NA is involved in ram-induced LH secretion as observed in other induced ovulators.

## Introduction

In all mammalian species that have been studied, ovulation is caused by the secretion from the adenohypophysis, of a large quantity of luteinizing hormone over a relatively short period: the LH surge. The LH surge is induced by an increase in secretion of the hypothalamic neuropeptide, gonadotrophin releasing hormone (GnRH). In species such as the sheep, the rat, the rhesus monkey and the human, the preovulatory secretion of GnRH and LH is tightly controlled by the oestradiol secreted from dominant follicle(s). In these species, the circulating concentration of oestradiol is elevated for several hours before a LH surge is first detected [1–5]. In ovariectomized ewes, rats and rhesus monkeys, the LH surge commences between 10 and 24 hours after exogenous oestradiol and independently of the mode of its administration: intramuscular [6], intravenous [7] or by sub-cutaneous implant [8–10].

In “induced ovulators”, such as the rabbit, the cat and the ferret the increases in GnRH and LH are also preceded by a pre-ovulatory increase in the concentration of oestradiol [11–14] but this rise alone is not sufficient to induce normal LH surges and ovulations [15–17]. These surges are only induced if the females are mated [18] although in some species (e.g. mink) pairing with a male is a sufficient stimulus [19–20]. The noradrenergic system has a central role in this phenomenon. In the rabbit and ferret, mating activates noradrenergic neurons [21–22]; and the extracellular concentration of noradrenaline in the mediobasal hypothalamus increases rapidly just prior to GnRH [23]. This increase can be reduced by a α1 antagonist administered into the arcuate nucleus [24]. Conversely, mating alone has a very limited effect on the induction of a LH surge without the sensitizing effect of oestradiol. For example mating alone induced an LH surge in only in 1 of 10 ovariectomized does [25].

In sheep, reproduction is seasonal and ewes stop cycling as day length increases and the ewes enter a season of anoestrus. During anoestrus their ovaries secrete very little oestradiol, there are no spontaneous pre-ovulatory LH surges and thus, the ewes do not ovulate. The introduction of a sexually active ram into a group of seasonally anoestrous ewes will induce an immediate increase in the pulsatile secretion of LH in close to 100% of ewes [26] and in a variable proportion of ewes, initiate a sequence of physiological events that culminate in a LH surge and ovulation [26–28]. This socio-sexual stimulation is often referred to as the “ram effect”. In most ewes this male-induced LH surge is preceded by a sustained increase in the plasma concentration of oestradiol lasting between 8 and 56 hours [29] and is similar to the reproductive neuroendocrine events seen in cyclic ewes during the breeding season [3, 4].

However, there have been consistent reports that small numbers of anoestrous ewes stimulated by the “ram effect” have "precocious" LH surges, defined as a LH surge starting between 0 and 8 hours after the introduction of rams [30–31]. The causes of these early LH surges have not been studied. In our laboratory precocious LH surges were seen in about 15% Ile-de-France and Mérinos d’Arles ewes [26, 29] and as happens with “normal” LH surges, they induced ovulation [26]. The time interval 0–8 hours is much shorter than that observed when the LH surge is induced with exogenous oestradiol [6, 9]; these LH surges occur too soon to be explained by the normal oestradiol-induced positive feedback mechanism.

One explanation is that although these ewes had low concentrations of progesterone and were considered anoestrous, they were on the verge of ovulating and that the precocious LH surge was simply a spontaneous LH surge that had been induced by a normal oestradiol-induced positive feedback just before or at the time of the “ram effect”. If this is so, then the circulating concentrations of oestradiol in these ewes should already be elevated at the time of introduction of the rams. An alternative explanation is that in these animals, the LH surge was induced by the contact with the ram, by a mechanism different from the classic oestradiol-induced positive feedback mechanism and perhaps closer to the mechanism responsible for the LH surge in induced ovulators. In fact several authors have suggested that the “dualistic” concept of ovulation as either spontaneous or induced is an over simplification and that the neural circuitry underlying induced ovulation also exists in species that ovulate “spontaneously” [32–33]. Some authors even suggest that induced ovulation is the ancestral mode of pre-ovulatory LH secretion [34]. In sheep contact with a sexual partner is known to have profound effects on the timing of reproductive events at all stages of reproductive life; it hastens puberty [35], induces ovulation during seasonal anoestrus [28] or lactational anoestrus [36] and modifies the latency of the LH surge during the breeding season [37]. In one study this effect was observed in oestradiol-treated ovariectomized ewes and is therefore the result of a direct stimulation of the hypothalamo-hypophyseal complex that does not involve ovarian feedback [38]. The pathway involved is not known but, increases in the extracellular concentrations of noradrenaline were detected in the posterior part of the preoptic area of ewes exposed to a sexually active ram and to a lesser extent, to his odor [39]. This supported the possibility of a role of noradrenaline in male-induced LH secretion in anoestrous sheep.

The aim of this study was to determine the concentrations of oestradiol in jugular venous plasma of anoestrous ewes immediately before and after the “ram effect” and to compare these patterns of oestradiol secretion in ewes with precocious and normal LH surges in response to the “ram effect”. In a second part we aimed to determine if noradrenergic neurons were activated during the “ram effect” and if by modulating this system, we could modify the LH response of ewes to the “ram effect” or to ram odor.

## Materials and Methods

The ewes were housed indoor on straw bedding under natural lighting in an enclosed building situated 800m from the rams building were the rams kept and were isolated from all contact with rams until the “ram effect” or the beginning of the experiments. They were always handled by staff that had no contact with the rams or their odours. They were fed a maintenance diet of hay supplemented with concentrate and with free access to water. The experiment was carried out in accordance with French and European regulations on the care and welfare of animals in research and with the authorization of the French Ministry of Agriculture (permit N° 006259) and the approval of the local ethics committee (permit N° 2012-01-2, comité d'éthique en expérimentation animale Val de Loire", N°19).

### The Relationship between the ram-induced LH surge and oestradiol

#### Experiment 1

**Fig 1:**
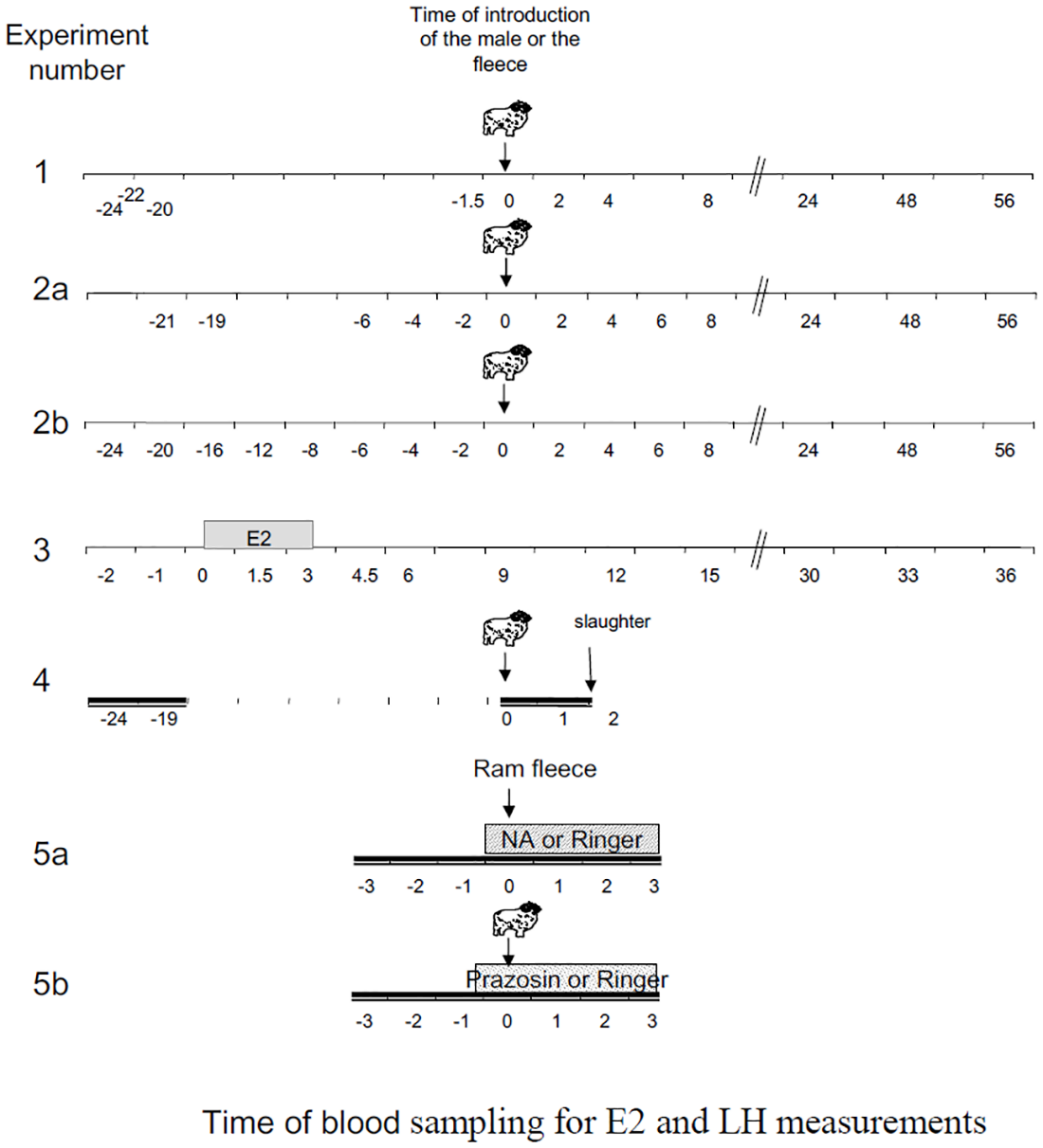
Schematic representation of the experimental design showing the time of collection of the samples that were used in the different experiments to measure oestradiol concentrations (Exp 1 and 2), and LH concentration (All experiments). Double time lines indicate that the samples were collected every 15 min.

The plasma samples analyzed in experiment 1 (the “precocious LH surge” study) were selected from a set of samples from another study the results of which have already been published [26]. In the published experiment we studied the pattern of plasma LH after the “ram effect” in adult sexually experienced Ile de France (IF) and Mérinos d’Arles (M) ewes some of which had “precocious LH surges”. Ewes were only used once and rams were always introduced at 10.00 am. In experiment 1, sets of samples from ewes with precocious LH surges (“precocious” n = 6 for each breed) were compared with sets of samples from ewes with a normal a LH surge (“normal” IF; n = 7 and M; n = 8). A “precocious” LH surge was defined as one starting between 0 and 6 hours after the introduction of rams and a “normal” surge was defined as one starting between 16 and 28 hours after the introduction of rams. The characteristics of the LH surge in these two groups are presented in Table 1. A third group consisting of ewes that had no evidence of an LH surge starting before the end of the experiment at 56h (“late LH surge” IF; n = 4 and M; n = 1) was also included. The concentration of oestradiol was determined in plasma samples collected 24, 22, 20, 1.5 and 0h before the introduction of rams and at 2, 4, and 8h after the introduction of rams as summarized in Fig 1. Blood samples were also collected once a week for 2 weeks before the “ram effect” for progesterone (P4) analysis to ensure that all experimental ewes were anoestrus [26] and then once a day after the ram effect to detect ovulation [26]. Details of the blood specimen and processing procedures have been published [26].

**Table 1:** Characteristics of the LH surge in the animals selected for study.

Experiment	Group	N	Time to onset of LH surge (h)	Duration of the LH surge (h)	Maximum concentration of LH (ng/mL)	P4 increased (d)
**Exp 1**	**“precocious”**	12	2.17± 0.47	14.83±0.62	33.25±3.98	3.5±1.25
	**“normal”**	15	17.87±1.16	15.07±1.22	28.50±1.57	4.0±2.0
	**“late”**	5	> 56		5.55±3.51	
**Exp 2**	***Year 1***					
	**“precocious”**	4	1.50±0.50	17.50±2.22	24.87±7.54	4.0±0.25
	**“normal”**	15	21.07±1.22	15.73±0.88	37.76±4.75	4.0±0
**Exp 2**	***Year 2***					
	**“precocious”**	4	1.06±0.36	18.94±1.86	35.68±2.36	3.0±0.50
	**“normal”**	11	19.27±0.67	18.91±1.86	36.13±3.03	4.0±0

The LH data are expressed as mean ± SEM and the P4 data as median ±interquartile. The onset of the surge was defined as the time when plasma LH first rose to a concentration that was more than 3 standard deviations above the baseline that preceded a sustained increase in LH of at least 4 hours and with at least one value above 10ng/mL [9]. The surge was considered to finish when the concentrations fell to below 10% of the maximum concentration. The latency of P4 increase was defined as the time of first observed concentration of P4 that was more than 3 standard deviations above the baseline.

#### Experiment 2

A second experiment (the “follow-up” study) was designed *de novo* to confirm and extend the results of Experiment 1. The experiment used 30 mature anoestrous Ile de France ewes. They were subjected to the “ram effect” at the end of anoestrus (July). The experiment was repeated the following year with the 28 ewes that were still alive. Two days before the experiment a catheter was introduced in the jugular vein for blood sampling and ewes were randomly distributed into small pens containing 5 ewes per pen. For the ram effect, we introduced into each pen, one ram from a group of 10 sexually active Ile de France rams from the UEPAO breeding flock that was used for the whole experiment. To make the stimulation as homogenous as possible in the different years, to minimize individual effects of rams and to maintain their levels of sexual activity they were changed regularly. After the 56h period of serial bleeding the ewes were reunited as a single group with 2 rams that were changed every week for 4 weeks. Ewes and rams were then separated until the next year. Blood samples collected between 0 and 56h after the introduction of the rams were analyzed for LH. These data were then used to identify and select ewes with a “precocious” LH surge (n = 4 each year, 2 of which had “precocious” surges in both years) or ewes with a “normal” LH (n = 15 year 1 and n = 11 year 2, 7 were the same on both years) as defined for experiment 1. Characteristics of the LH surges and progesterone increases in these ewes are shown in Table 1. The samples from these ewes were then analyzed for oestradiol as summarized in Fig 1: Year 1; 21h and -19h before the introduction of rams and Year 2; -24h, -20h, -16h, -12h and then for both years every 2 hours between -6h and 8h relative to the introduction of rams. As in experiment 1, blood samples were also collected to measure progesterone and detect ovulation [26].

#### Experiment 3

A third experiment (the “low E2” experiment”) was done to determine if a small increase in the circulating concentrations of E2 lasting three hours could by itself induce a LH surge in anoestrous ewes. Eleven intact anoestrus ewes were implanted subcutaneously for 3 hours with a 3 cm sealed silastic tube containing oestradiol a technique known to produce increases in oestradiol concentrations in plasma in the range of those observed in experiment 2 [40]. This experiment was carried out at the end of anoestrus using ewes from experiment 2 that had had at least one “precocious” LH surge in the previous years and were therefore presumably more sensitive to oestradiol (7 ewes) or that had had only “normal” surges (4 ewes). These ewes were kept without contact with rams for a minimum of 3 months.

Samples were collected at hourly intervals and just before oestradiol implantation (-2, -1 and 0 hours) then every 1.5 hours for the first 6 hours after implantation and finally every 3 hours until 36 hours as illustrated in Fig 1 to measure LH concentrations. E2 concentrations were measured in samples collected on 5 ewes (2 that had never had a precocious LH surge and 3 that had) 2 hours before and just before implantation (time 0), during implantation (time 1.5 and 3) and after implant withdrawal (time 4.5h). Details of the blood sampling and processing procedures have been published [26].

### The role of the noradrenergic system in the male-induced LH Surge

#### Experiment 4

In a fourth study (the “histological” study), noradrenergic neurons activated during the “ram effect” were identified using a double labelling procedure to detect presence of cFos a known marker of neuronal activation and tyrosine hydroxylase (TH) the rate limiting enzyme for synthesis of noradrenaline.

(a) Sample collection

During anoestrus, 11 ewes were habituated to human contact for two weeks before the experiment. Blood samples were collected through an indwelling jugular venous catheter as previously described [26] every 15 min for 5 hours (Fig 1) to measure basal LH secretion. The following day ewes were divided into two random groups and treated either by the introduction of a sexually experienced adult ram into their pen (male-exposed group, n = 5) or by continuing in isolation from rams (control group, n = 5). The 2 groups were handled by different staff to avoid any contamination of the control group with male odour. Ninety minutes after the introduction of the ram the ewes were terminated by decapitation by a licensed slaughter man following a protocol agreed by the ethics committee. Blood samples were collected every 15 min during the last hour to determine the endocrine response to stimulation. The heads were then immediately perfused through both carotid arteries with 2L of 1% sodium nitrite in phosphate buffer (0.1 M, pH 7.6) and 4L of cold 4% paraformaldehyde (in phosphate buffer). The brains were then removed intact, post-fixed for 24h in 4% paraformaldehyde and then left in phosphate buffer containing 30% sucrose and 0.1% sodium azide. The fixed brains were cut transversely into three approximately equal blocks. Free floating sections of the posterior block (40μm) were cut on a freezing microtome (Leica, Paris, France) and stored in cryoprotectant (NaCl 9%, polyvinyl pyrrolidone 10%, saccharose 30%, ethylene glycol 30% in phosphate buffer 0.1 M pH 7.4) at 4°C. Every 10th section was stained with Cresyl violet to allow identification and delineation of noradrenergic brain areas according to Tillet and Thibault [41].

(b) Immunohistochemistry

For each animal, the noradrenergic nuclei were identified and sections selected to contain comparable structures among ewes (5 sections per ewe for A1, A2, A6, A7 and 3 for A5 nuclei). The sections were first stained for Fos [42] using an affinity-purified rabbit polyclonal antibody raised against the Fos protein (Ab-2, PC38, Oncogene Research Products, Calbiochem, San Diego, CA, USA, diluted 1/60,000 in PBS-TA-BSA, 2 days, 4°C) and peroxidase-anti-peroxidase complex solution (Jackson Immunoresearch, West Grove, PA, USA), diluted in 1/1,000 in PBS-BSA, 4°C and visualized using 3-3’diaminobenzidine tetrahydrochloride (DAB, Sigma Chemical, St Louis, MO, USA) intensified with 0.3% nickel ammonium sulphate. The sections were then rinsed in phosphate-buffered saline (PBS, 10% phosphate buffer 0.1 M, pH 7.4, 0.9% NaCl in distilled water) and stained for TH [43] using a mouse monoclonal antibody raised against TH (Chemicon International) followed by diluted 1/1,000 and a peroxidase-antiperoxidase conjugated to sheep anti-mouse antibody (Jackson Immunoresearch) diluted 1/500 and then visualized with 3-3’diaminobenzidine tetrahydrochloride (Sigma Chemical, St Louis, MO, USA).

(c) Quantification of immuno-labelling

Quantification of Fos positive cells (Fos-IR) was performed using an image analysis system equipped with software to analyze cell-count data (Mercator, Explora Nova, and La Rochelle, France). To count Fos positive cells a microscope with a motorized stage and a video camera was connected to a computer with a color monitor. Using the program we established parameters of size, shape and threshold for grey scale to characterize Fos-IR positive cells [42]. The average background grey scale was automatically estimated for every section and subtracted from the original image before the software identified cells meeting the established parameters as Fos-IR positive cells. Each section was also examined visually and any suspect objects (e.g. dust) were erased manually. For counting TH-IR and Fos-IR/TH-IR positive cells, a manual system (Biocom, Paris, France) was used. Images were viewed on a monitor by an observer blind to the treatment used and who identified each immunoreactive cell on the screen. Because A6 and A7 overlap they were counted together as LC-A7.

#### Experiment 5

In a fifth study (the “pharmacological” study) we determined if the local infusion of noradrenaline could enhance the short-term LH response evoked by a sub-stimulating ram cue made of a handful of ram fleece (experiment 5A) or alternatively block the short-term LH response evoked by exposure to a sexually active ram with the local administration of the α1 adrenergic antagonist Prazosin (experiment 5B).

Two to 4 weeks before the start of the experiment, 15 mature Ile-de-France ewes were fitted bilaterally, with guide cannulae directed at the posterior preoptic area using a technique which combined a stereotaxic method with lateral and frontal radiography [44]. The procedures were carried out under general anesthesia induced by the intravenous injection of thiopental (1g per ewe; Nesdonal, Specia Rhone Poulenc, Paris, France) and atropine sulfate (20mg per ewe; Lavoisier, Paris, France) and maintained by closed-circuit halothane (Bélamont, Neuilly, France). Full aseptic precautions were taken throughout. After surgery, ewes were injected with 5ml of Dexamethasone per ewe (Diurizone, Vetoquinol, Lure, France) daily for 3 days. The ewes were allowed a minimum of 2 weeks recovery time and during their recovery the ewes were habituated to handling and the presence of humans.

The day before the experiment, noradrenaline (Research Biochemical international N-112 Natick USA) was dissolved in 1 mL HClO_4_ 0.25M. On the day of the experiment noradrenaline, and crystalline Prazosin (Sigma P7791 Saint Quentin Fallavier France) were dissolved in ringer’s solution (pH6.5) to a final concentration of 100ng/mL for noradrenaline and 100μg/mL for Prazosin. The dose of noradrenaline was chosen to produce local concentrations in the range of those measured in a previous experiment [39] and the dose of Prazosin was chosen from preliminary trials. Noradrenalin, Prazosin or their solvent were infused by retrodialysis at a rate of 2μl/min using microdialysis probes (Mab 6 Microbiotech Sweden, 5mm membrane) inserted into the guide cannulae. Infusion started half an hour before exposure to a handful of ram fleece that will increase LH increase in some but not all ewes (experiment 5A), or a sexually active ram that will increase LH in all ewes (experiment 5B). The fleece used was a mixture of fleeces from 10 rams (Ile de France and Romanov) different from those used in the experiment and that had been collected during the breeding season and stored at -20°C. The rams used in this experiment were from a group of sexually active rams from the station breeding flock and were changed regularly among pens during the 3 hours to minimize individual effects of the rams and maintain their level of sexual activity. A delay of half an hour was allowed for the test reagents to be transferred from the syringe to the dialysis membrane at the brain site. Experiment 5A took place in March and April and experiment 5B in July. In each experiment ewes acted as their own controls and the order of infusion of the treatment solution and the control solution was random at least one week apart. Any ewe that showed evidence of luteal activity (a progesterone concentration above 0.5 ng/mL) was not used. Plasma samples were collected every 15 min for 3 hours before and 3 hours after treatment (Fig 1).

The concentrations of progesterone in plasma were monitored weekly during the experiment and infusions were carried out only on ewes confirmed as anoestrus that is with progesterone concentrations <1ng/mL for a minimum of 2 weeks. In both experiments (5A & 5B) 11 anoestrous ewes were available, 7 ewes were used in both experiments and 4 ewes were used in only 1 experiment.

At the end of experiment all the females were killed. Free-floating frontal sections (40μm thick) were cut and stained with cresyl violet to facilitate histological identification of probes location as described for experiment 4.

### Assay of Oestradiol

The concentrations of oestradiol in jugular venous plasma were determined using the HRP- oestradiol DIASource immunoassay ELISA kit (E2-EASIA / KAP0621; DIASource immunoassay SA, Louvain la Neuve, Belgium), adapted for the detection of oestradiol in ovine plasma [29]. The sensitivity of the assay is 0.39 pg/mL and its detection limit 0.78 pg/mL. The intra-assay and inter assay coefficients of variation were: 16.6% and 15.3% (at 0.46 pg/mL), 11.9% and 11.7% (at 0.80 pg/mL) and 4.6% and 4.7% (at 5.13 pg/mL).

### Assay of Progesterone

The concentration of progesterone was measure using an ELISA [26]. The sensitivity of the assay was 0.2 ng/mL and the intra-assay and inter-assay coefficient of variation were 6.8% and 8.1% for a reference at 1.5 ng/mL and 6.6% and 10.3% for a reference at 2.5ng/mL.

### Assay of LH

The concentrations of LH in jugular venous plasma were determined using a radioimmunoassay [45]. The assay sensitivity was 0.16 ng/mL standard 1051-CY-LH (equivalent to 0.31 ng/mL NIH LH-S1). The intra-assay and inter assay coefficients of variation were: 4.4% and 10.3% respectively.

The onset of the LH surge was defined as the time of the first observed concentration of LH that was more than 3 standard deviations above the baseline and preceding an increase in LH of at least 4 hours and with at least one value above 10ng/mL [9, 46]. LH pulses were identified as an increase in LH concentrations >3SD above the baseline defined as the mean concentration before ram introduction. An animal was classified as having a short-term LH response if the number of LH pulses during the 3 hours after stimulation was superior to the number of pulses during the 3 hours before stimulation. The amplitude of a LH pulse was calculated as the difference between the maximum concentration of LH in the pulse and the concentration of LH in the sample before the start of the pulse.

### Statistical analyses

Statistical tests were carried out using Statistica version 10 (Statsoft Inc.). The data involving oestradiol measured over time were analyzed using a mixed model ANOVA run under the general linear model with time as a repeated measure. Data from each year in experiment 2 were analyzed separately. Paired comparisons within treatments were carried when appropriate, using the Bonferroni correction. The data involving LH measured over time were also analyzed using an ANOVA with repeated measures.

The mean density of immunoreactive cells was calculated for each region of each animal and the overall median and inter-quartile values calculated for the two groups. Because the mean densities were not normally distributed, statistical comparisons were carried out using nonparametric tests: The Kruskal and Wallis test followed by the Mann Whitney U test to compare groups or the Friedman tests to compare the density among nuclei in each group.

A ewe was considered as responding if the frequency of LH pulse was at least 1 pulse per 3 hours greater after stimulation than before. The proportions of ewes showing increased pulsatile LH activity were compared by χ² tests. The LH pulse frequency and the amplitudes of LH pulses before and after stimulation and during the treated versus control session were compared using Wilcoxon test.

Differences were taken as statistically significant at p <0.05 and as a trend at p >0.05 but <0.10.

## Results

### The “precocious” surge study

**Fig 2:**
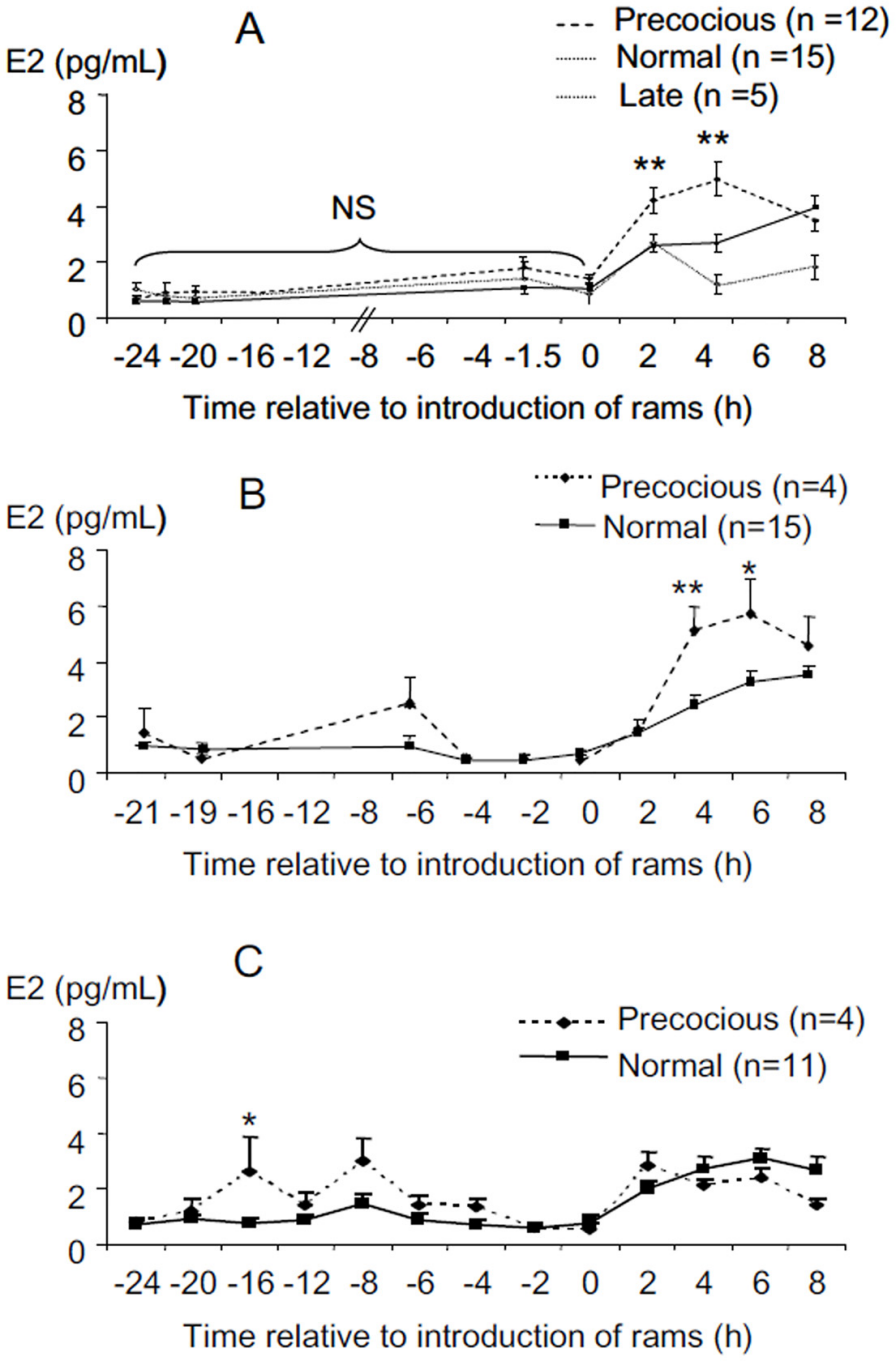
Changes in oestradiol concentrations before and after ram introduction in anoestrous ewes who presented LH surges at different time after ram introduction. "precocious": ewes who presented a LH surge within 4 hours after male introduction; "normal": ewes presented a LH surge between 16 and 28 hours after the introduction of rams; "late": ewes who did not present a surge before 56h. A exp. 1, B exp. 2 year 1, C exp. 2 year 2. Data are expressed as Mean ± SEM. * p<0.03, **p<0.01 compared to “normal” at the same time.

The duration and amplitude of the LH surge did not differ between "precocious" and "normal" surges (Table 1). All the LH surges resulted in ovulation as shown by increases in P4 concentrations and the latency of this increase did not differ (Table 1). However, the concentrations of oestradiol in ewes with "precocious", "normal" or "late" LH surges did differ (Fig 2A). There was a significant effect of time (p <0.0001) and of the type of response (p = 0.029), but no effect of breed (p = 0.890) so the data of the 2 breeds were pooled. There was a trend towards a significant interaction between the type of response and time (p = 0.064). Paired comparisons showed that the concentrations of oestradiol in the samples taken at 2h, 4h and 8h after the introduction of the rams differed significantly from of those taken before the introduction of rams (all comparisons p <0.001). Before the introduction of rams, there were no significant differences in the mean concentration of oestradiol among the types of response ("precocious”, 1.15 ± 0.15 pg/mL; "normal", 0.82 ± 0.08 pg/mL and "late" 1.06 ± 0.31 pg/mL). Following the introduction of rams the concentrations of oestradiol at 2h and 4h, in the "precocious" group (2h: 4.26 ± 0.27 pg/mL and 4h: 5.03 ± 0.65 pg/mL) were significantly greater than those in "normal" (2h: 2.60 ± 0.27 pg/mL, p <0.005 and 4h: 2.65 ± 0.32 pg/mL, p <0.001) and at 4 h than the "late" responders (1.17 ± 0.36 pg/mL, p <0.002).

### The “follow-up” study

The concentrations of oestradiol in ewes with "precocious" LH surges compared to ewes with “normal” LH surges are illustrated in Fig 2B and 2C. In both years, the concentrations of oestradiol at the time of ram introduction (time 0) were not significantly different in ewes with "precocious" and “normal” LH surges and increased after the introduction of the ram.

The global analysis showed a significant effect of time for each year (p<0.0001) and an interaction between time and type of response (year 1 p = 0.002, year 2 p = 0.0006) but no significant effect of type of response (p = 0.21 and 0.22 for years 1 and 2).

When the periods before and after the “ram effect” were analyzed separately, a significant effect of the type of response was observed. During year 1 type of response had no significant (P = 0.28) effect before the "ram effect" (Mean oestradiol concentrations = 1.01±0.28 and 0.75±0.11pg/mL for “normal” and “precocious”). But type of response had a significant effect after the "ram effect" (p<0.047). Paired comparisons showed significant differences at 4 and 6 hours after the ram effect between the “precocious”and “normal” groups (Fig 2B.; +4h: 5.1±0.87 versus 2.43±0.32 pg/mL, p<0.006 and +6h: 5.66±1.25 versus 3.24±0.39 pg/mL p<0.027).

In the second year a significant effect of the type of response was observed before the “ram effect” (p<0.03) and paired comparison showed that oestradiol concentration at -16h before the “ram effect” was significantly greater in the “precocious” group (Fig 2C; 2.61±1.27 versus 0.82±0.16 pg/mL; p<0.03) and approached significance at -8h (3.02±0.81 versus 1.30±0.35 pg/mL; p = 0.056). The mean concentrations of oestradiol after ram introduction were higher than before but, did not vary between type of response (“precocious”: 2.20±0.10, “normal”: 2.49±0.37 pg/mL).

### The “small E2” study

**Fig 3:**
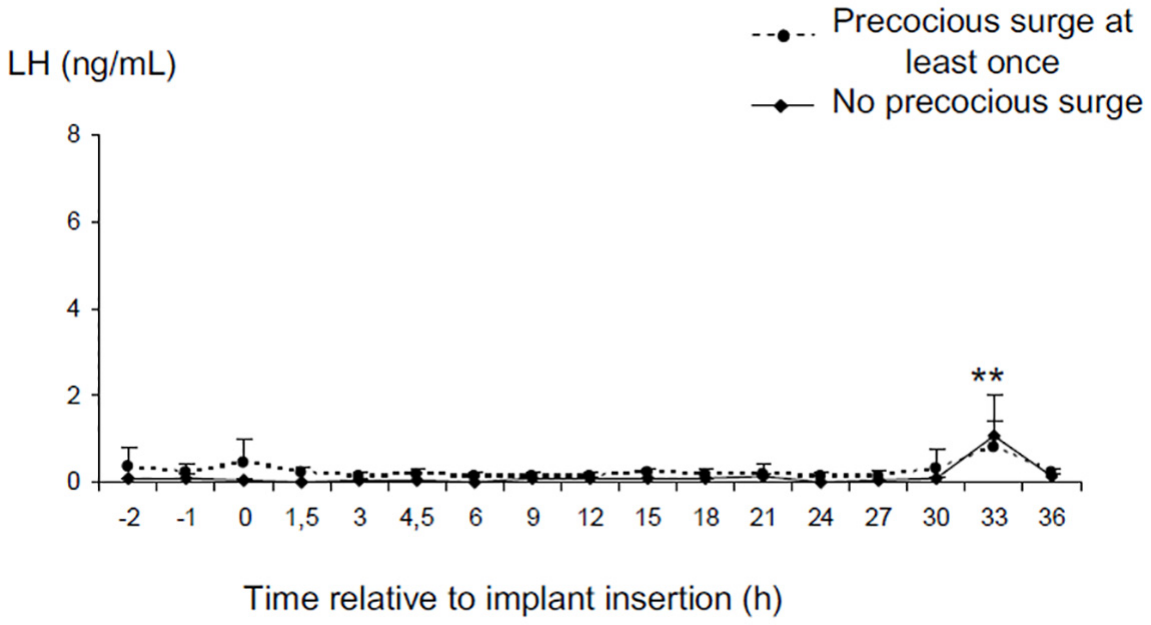
Changes in LH concentrations before and after insertion of a 3 cm silastic oestradiol implant for 3 hours. (small E2 study). Data are expressed as Mean ± SEM **p<0.01 compared to other times.

Data from one ewe was discarded because the oestradiol implant could not be retrieved. Insertion of the oestradiol implant for 3 hours increased the plasma concentrations of oestradiol from the detection limit of the assay (0.51±0.3 and 0.65±0.25 respectively at time -2 and 0) to 1.0± 0.24, 2.29±0.96 and 5.91±4.53 pg/mL respectively 1.5h 3h and 4.5h after implantation. These implants did not stimulate a LH surge in any ewe (Fig 3). However, LH concentrations changed with time (p<0.001). The mean E2 concentration fell from 0.25± 0.06 ng/mL before implantation to 0.13±0.03 ng/mL while the implants were in place (p>0.1) and to 0.11±0.03 ng/mL in the following 3 hours (p>0.1) and then increased at 33h after insertion of the implant (0.88± 0.35 ng/mL p<0.01 compared to the concentrations before during and just after E2 implantation). There was no significant difference between groups with a history of “precocious” LH surges compared to those with no history of “precocious” LH surges. By contrast the ewe with an implant present for the whole experiment, had a LH surge that started 18 hours after E2, lasted for 15 hours and had a maximum LH concentration of 13.6 ng/mL (Fig 3).

### The “histological” study

The mean LH concentration the day before stimulation did not differ between groups (p = 0.16). All ewes exposed to a ram but none of the control ewes exhibited a significant increase in mean LH concentration during the 90 min stimulation compared to the level the day before (Ram: 1.15±1.6 ng/mL vs 0.55±0.03, p<0.01; control: 0.40±0.09 vs 0.37± 0.09 ng/mL, p = 0.64).

**Fig 4:**
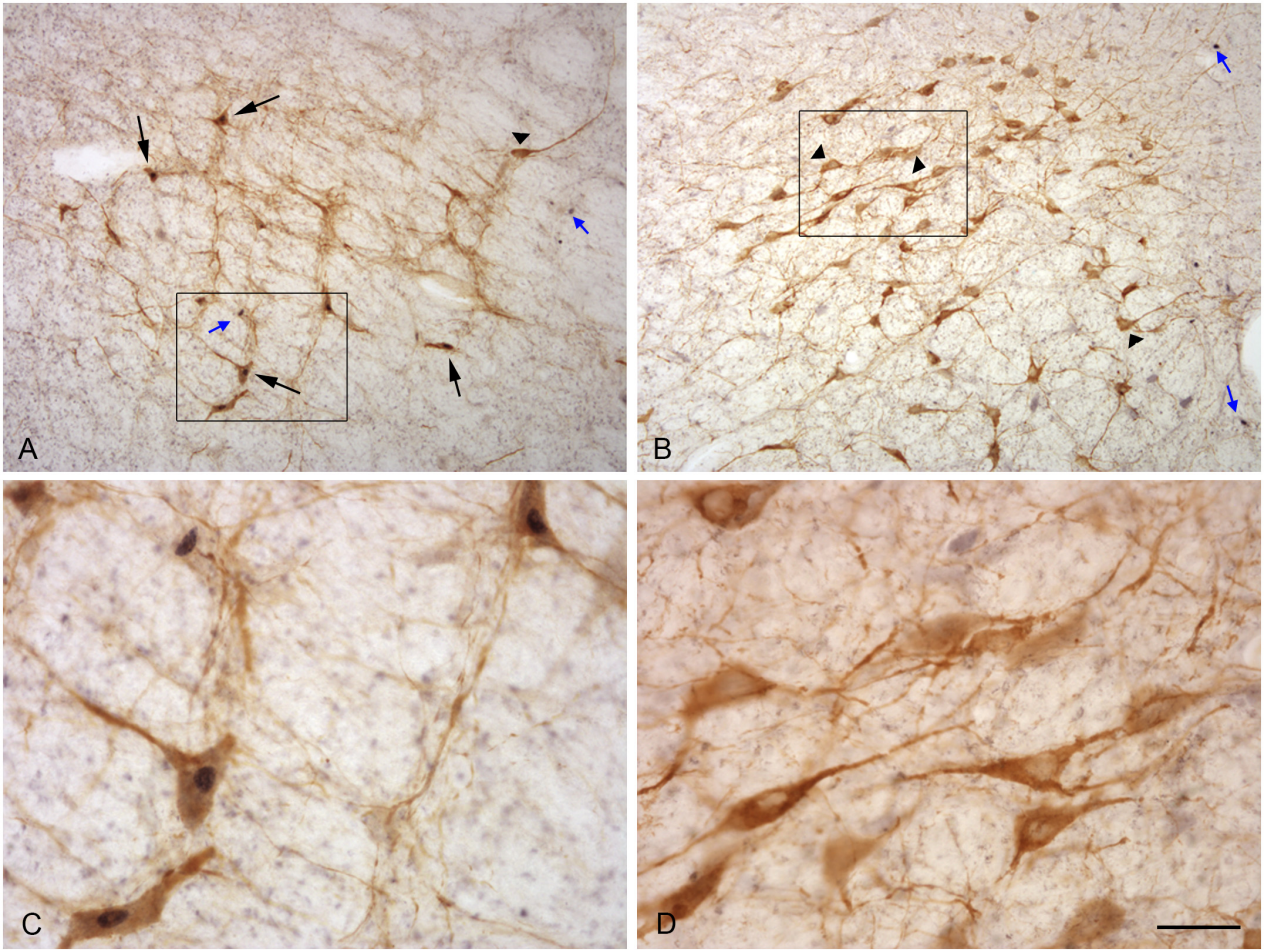
Photographs illustrating neurons expressing Fos and TH proteins in the A1 (A, C) and A7 (B, D) nucleus of a female exposed to a ram. C and D are details of the area shown in the rectangle. Small blue arrows: Fos alone; black arrows: Fos protein in a TH neuron; arrow heads: TH without Fos protein. Scale bar = 160μm for A and B and 40μm for C and D.

The TH immunoreactive cells were characterized by a brown precipitate in the cytoplasm and could be identified in all the locations where noradrenergic neurons have been described in sheep [41].The fos immunoreactive cells were characterized by a dense black precipitate in their nuclei that appeared as a round black structure easily detected by our image analysis system and very similar to the labelling we had previously observed in hypothalamic and olfactory structures [42]. Double labelled cells could be identified by the presence of a dark nucleus in a brown cytoplasm (Fig 4).

**Fig 5:**
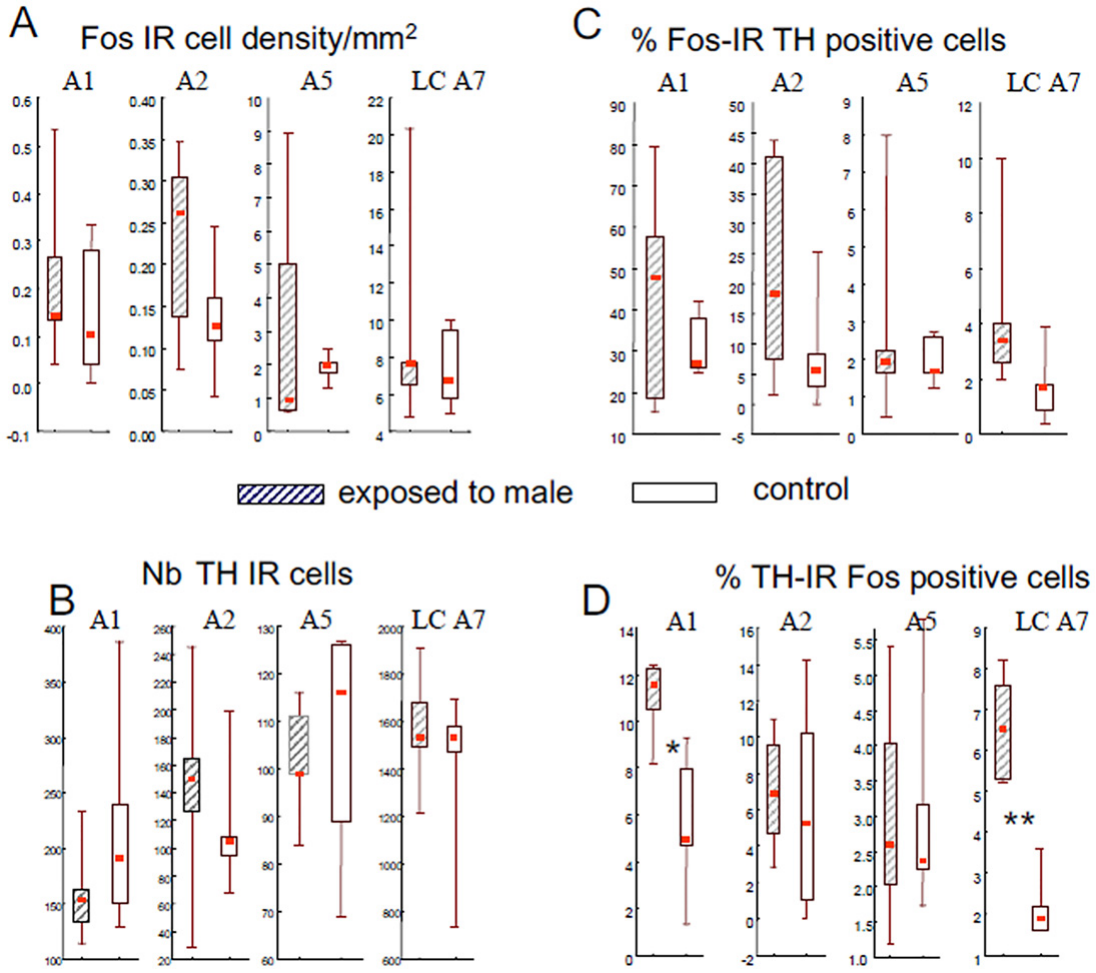
Box Plot representations of the density of Fos-IR neurons (A), the number of TH-IR cells counted (B), the proportion of Fos-IR cells also containing TH-IR (C) and proportion of TH-IR cells also containing Fos-IR (D) in the different noradrenergic nuclei (A1, A2, A5, LC-A7) in adult Ile de France ewes exposed to a ram (hatched boxes) or to a control situation (empty boxes). The bottom and top of the boxes are the first and third quartiles, the red square inside the boxes is the median and the end of the whiskers are the minimum and maximum of all the data; * p<0.02, ** p<0.01 compared to the control group. Because of the differences in the number, density and proportions among the different nuclei the scales are different for the different nuclei.

The density of Fos immunoreactive cells was significantly different between monoaminergic nuclei (p<0.001 in both groups) and was higher in LC-A7 complex than in the other nuclei (p< 0.005) but did not differ in the control or treated ewes (Fig 5A). Similarly, the number of TH immunoreactive cells did not differ between females exposed to ram and to the control situation (Fig 5B). The proportion of Fos immunoreactive cells that were also TH immunoreactive did not differ between groups of females although in the LC A7 nucleus it tended to be higher in ewes exposed to rams (p = 0.060, Fig 5C). By contrast the proportion of TH immunoreactive cells that were also immunoreactive for Fos differed between groups and the proportion of double-labelled cells in A1ventrolateral medulla nucleus (p<0.02) and the locus coeruleus complex (p<0.01) was higher in ewes exposed to a ram than in the controls (Fig 5D).

### The “pharmacological” study

**Fig 6:**
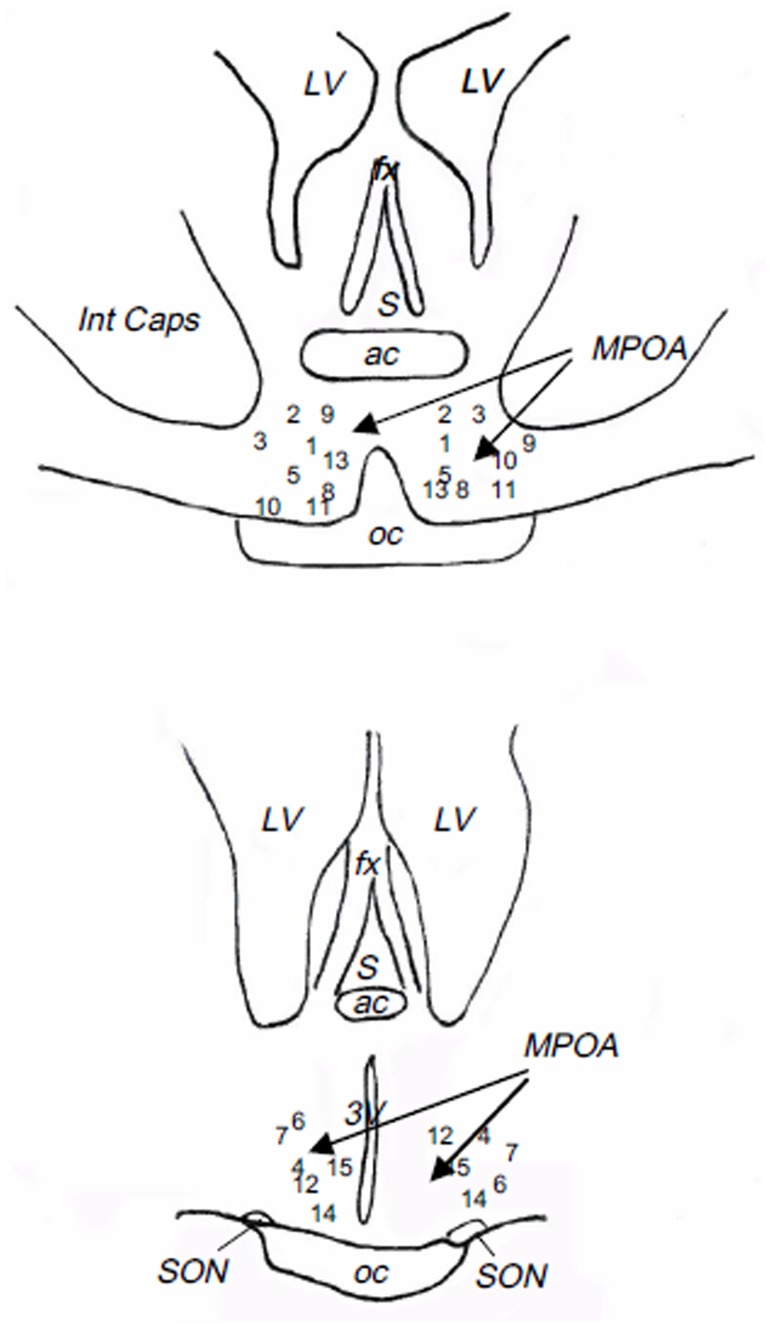
Schematic representation of the localization of the tip of dialysis probes used in the “pharmacological study”. Numbers correspond to the different animals. ac: anterior commissure, fx: fornix, Int Caps: Internal Capsule, LV: lateral ventricle, MPOA: medial preoptic area, oc: optic chiasma, S: septum, SON: supraoptique nucleus, 3V: 3^rd^ ventricle.

As shown in Fig 6, the ends of the guide cannulae were all located in the posterior preoptic area (Anteroposterior coordinates = 32 from the Richard’s atlas [47]).

**Fig 7:**
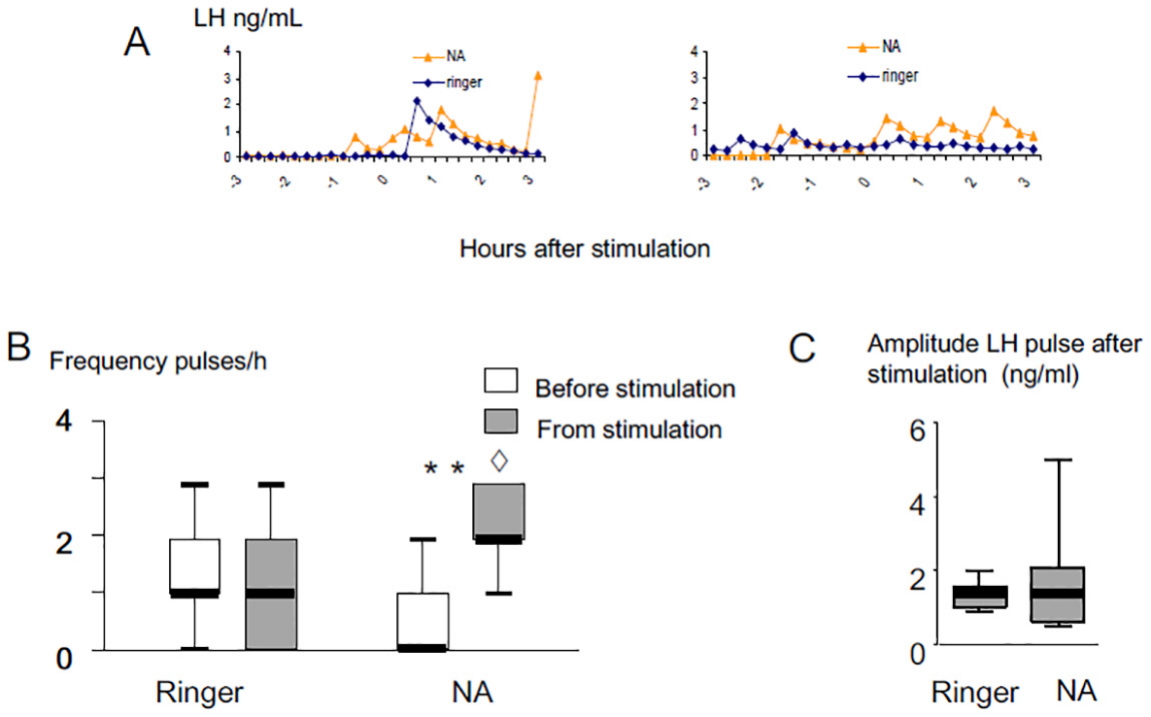
Effect of infusion with noradrenaline (100 ng/mL) on LH secretion induced in anoestrous Ile de France ewes by exposure to ram fleece. A: Representative LH profiles in 2 ewes; B: Box Plot representations of the effect on pulse frequency, C: Box Plot representations of the effect on pulse amplitude. The thick horizontal lines depict the median. ** Different from noradrenaline before the stimulation p<0.003, ◊ different from ringers after stimulation p<0.02.

Examples of LH profiles in response to ram fleece in ewes infused with noradrenaline or ringers are shown Fig 7A. The infusion of noradrenaline into this region increased the proportion of ewes responding to ram odor (9/11 versus 5/11; p = 0.03). The frequency of LH pulses before stimulation was not different between ewes infused with noradrenaline or ringers (p>0.1). But after exposure to ram odor the frequency of LH pulse was higher than before in ewes infused with noradrenaline (Fig 7B; p<0.003) and higher than after in ewes infused with ringers (p<0.02). By contrast exposure to ram odor for 3 hours had no significant effect in control ewes. Noradrenaline treatment did not affect the amplitude of LH pulses after exposure to ram odor (p = 0.12; Fig 7C).

**Fig 8:**
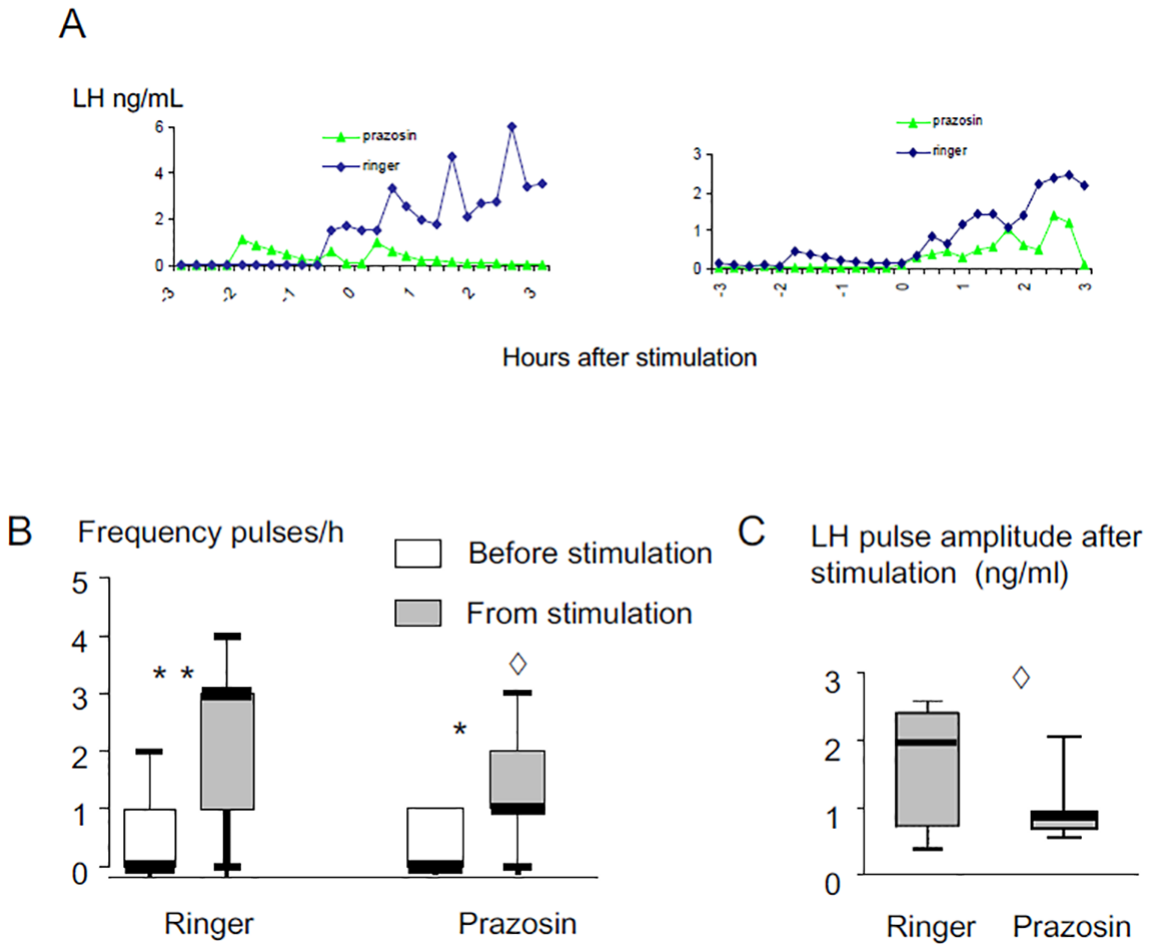
Effect of infusion with Prazosin (100 μg/mL) on LH secretion induced in anoestrous Ile de France ewes by exposure to a ram. A: Representative LH profiles in 2 ewes; B: Box Plot representations of the effect on pulse frequency, C: Box Plot representations of the effect on pulse amplitude. * Different from before the stimulation p<0.02, ** different from before the stimulation p<0.003, ◊ Prazosin different from ringer p<0.04.

Examples of LH profiles in response to exposure to a ram in ewes infused with prazosin or ringer are shown Fig 8A. Prazosin did not affect the proportion of ewes responding to the “ram effect” (7/11 versus 8/11) and introduction of a ram increased LH pulse frequency in both ringers (p<0.003) and Prazosin-treated sessions (p<0.02; Fig 8B). However, the frequency (p<0.03; Fig 8B) and the amplitude (p<0.04; Fig 8C) of LH pulses following the “ram effect” were significantly lower after Prazosin treatment compared to the ringers sessions.

## Discussion

Mammals are traditionally considered as either spontaneous or induced ovulators. Sheep are spontaneous ovulators and the LH surge in cyclic ewes is driven by a large and sustained preovulatory rise of oestradiol [3, 5, 48]. During anoestrus there are no spontaneous LH surges. However, anoestrous sheep can be induced to ovulate by the sudden exposure to a ram. In the present study we show that some ewes have LH surges occurring within 4 h of the introduction of rams ("precocious" LH surge) that are not preceded by large increased concentrations of oestradiol. We also show that in anoestrous ewes, exposure to rams activated the noradrenaline neuronal populations of the locus coeruleus complex and the A1 nucleus and that we can modify the LH response to male socio-sexual cues by locally manipulating the noradrenergic system in the preoptic area. Together these results suggest that in anoestrous ewes two partly different neural circuits are involved in the ram-induced LH surge, the neural circuitry involved in the mechanism of the ram-induced “precocious” LH surge in anoestrous ewes being different from that for the LH surge induced by the classic oestradiol positive feedback mechanism. They also suggest that the noradrenergic system is involved in the LH response to male socio-sexual cues and extrapolating further, that “precocious” LH surges are a result of higher activation of the noradrenergic system possibly using a neuronal circuit partly similar to that in the mating-induced LH surge of induced ovulators.

In anoestrous ewes LH pulsatility is very low, ovaries secrete little oestradiol and no progesterone because ewes do no ovulate and do not have corpora lutea. However introduction of a sexually active ram will induce an immediate increase in the pulsatile secretion of LH in close to 100% of ewes, and in a lower and variable proportion of ewes a LH surge [26]. In most ewes this LH surge occurs 12 to 56 hours after the introduction of a ram [26–28, 30] and is preceded by a sustained increase in the plasma concentration of oestradiol lasting between 8 and 56 hours with concentrations above 2.5 pg/mL 4 hours before the surge [29]. This is similar to what happens in cyclic ewes during the breeding season; when the LH surge is preceded by an increase in the plasma concentration of oestradiol that normally lasts more than 12 hours [3, 4]. The present study showed that this was not the case in ewes with a “precocious” LH surge although the durations and peak concentrations of these “precocious” LH surges are not different from “normal” LH surges and they were followed by similar increases in progesterone concentration indicating that the ewes ovulated. In our study these “precocious” LH surges were not preceded by a large increase in oestradiol concentrations and oestradiol concentrations at the time of ram introduction when the “precocious” surges were starting were still low. So these ewes probably were not on the verge of a spontaneous ovulation at the time of exposure to rams. In all ewes, the concentration of oestradiol increased within 2 hours of the “ram effect”. This increase is undoubtedly the result of increased pulsatile secretion of LH, each pulse of LH stimulating the release of a pulse of oestradiol by the ovary [49]. In our study the immediate increase in oestradiol concentration was greater in ewes with “precocious” LH surge compared to those with a “normal” surge in exp. 1 and exp. 2A but surprisingly not in exp 2B. The fact that we did not see this difference in all 3 experiments suggests that this is not a prerequisite for a “precocious” LH surge.

Another possibility is that, in these “precocious” ewes, LH surges were induced by small and short increases in oestradiol concentrations that we could not detect. Indeed, in the second year of experiment 2 when samples were collected over a longer interval some ewes had episodic increases in oestradiol secretion resulting in significantly higher concentration of oestradiol 16 hours before ram introduction compared to ewes with a normal LH surge. Studies on ovariectomized ewes treated with oestradiol implants during the breeding season have shown that LH surges can be induced by a much shorter period of elevated oestradiol than normally occurs in cycling ewes. A LH surge was observed in 1/12 Suffolk ewes exposed to oestradiol for 7 hours [50] and in 3/10 Ile de France ewes exposed to oestradiol for 3 hours [51]. However in these studies the reported concentrations of oestradiol were higher than in our study (8pg/mLversus 2.6 and 3pg/mL, 16h and 8h before the “ram effect”). In our third experiment we showed that an increase in oestradiol concentration in the range of the increase observed in experiments 1 & 2 was not enough, by itself, to induce a LH surge although it did slightly modify LH secretion. So it is very unlikely that the small increases in oestradiol observed in our study were alone, sufficient to induce “precocious” LH surges using the same mechanism as for the "normal" surges. It is theoretically possible that long exposure to undetectable level of E2 may be sufficient to produce precocious surge but in all of our data we found no evidence that this is the case. We suggest that in our study the LH surge was induced by a combination of oestradiol priming and male stimulation in a similar way as occurs in induced ovulators.

In induced ovulators an increase in oestradiol induces receptive behavior and allows mating and vaginal stimulation produced by mating, to induce a LH surge [18]. The noradrenergic system has a central role in this male induced LH surge [14, 18]. In rabbits and ferrets mating stimuli activate noradrenergic neurons in the locus coeruleus and the brainstem [21, 22]. This leads to a rapid increase in the extracellular concentrations of noradrenaline in the mediobasal hypothalamus just prior to the increase in GnRH [23] and this increase can be reduced by α1 antagonists [24].

Anoestrous ewes are not sexually receptive and refuse all male courtship behavior so there can be no vaginal stimulation. But in a previous study we have shown that the extracellular concentrations of noradrenaline increased in the posterior preoptic area of anoestrous ewes exposed to a ram and to a lesser extent, to ram odor [39]. Here we show that as in induced ovulators, noradrenergic neurons from the locus coeruleus complex and from the brainstem were activated when anoestrous ewes were exposed to a sexual stimulus but without vaginal stimulation. In sheep as in other species, noradrenergic neurons project fibers to the preoptic area [52, 53]. So these neurons are the probable origin of the increase in noradrenaline concentration in the preoptic area caused by exposure to a ram. Interestingly the A1 nucleus that was activated in our study is also activated in ovariectomized ewes after treatment with oestradiol at a dose that will induce a LH surge [54] suggesting that the neural networks involved in spontaneous and ram-induced LH surges overlap to some extent. The relatively low proportion of TH cells that were Fos positive probably reflects the functional heterogeneity of these cell groups and the fact that noradrenaline is in involved in many aspects of brain function.

The high sensitivity of ewes towards a sexual partner is not surprising because during the breeding season an increase in noradrenaline was observed in the mediobasal hypothalamus when oestrous ewes were exposed to a picture of a ram’s face and to a lesser extent to ram odor [55] whereas in oestrous rats noradrenaline increased only after vaginal stimulation [56]. Furthermore, in some induced ovulators such as the mink mating is not necessary and pairing alone is provides sufficient sexual stimulation to induce ovulation [19, 20].

In our study we were able to increase the effectiveness of male odor by the local administration of noradrenaline into the preoptic area of anoestrous ewes or to reduce the pulse frequency and amplitude of the short-term LH response to the “ram effect” with a α1antagonist. These results suggest that in this context, noradrenaline has a facilitatory action on LH secretion in response to male socio-sexual cues. The effect of noradrenaline however was not total and we did not reduce the proportion of ewes showing a response to the ram. Noradrenergic receptors are present in high density in the preoptic area [57, 58]. The most likely explanation is that our treatment did not block all the noradrenergic receptors and that enough noradrenergic receptors were active to evoke a slight increase in LH pulse frequency in response to the ram.

Because of the sampling duration relative to ram or fleece exposure, the stimulatory effects of noradrenaline we observed are most likely on LH pulsatility. Although noradrenaline has been implicated in the control of LH secretion for several decades [59] its role in the pulsatile activity of GnRH neurons is complex and not completely understood [60]. In rodents some studies have shown that the administration of noradrenaline or of a α1 adrenergic agonist inhibits LH pulsatility [61] but the same effects have been observed by others after administration of a α1 antagonist [62]. The same results have been observed in sheep where the effects appear to vary with the steroid milieu. [63–64]. This has led authors to suggest that the role of noradrenaline is permissive and depends on the relative contribution of other neurotransmitters and neuropeptides [60]. However, LH pulsatility depends on a “pulse generator” situated in the mediobasal hypothalamus [65] that is 3mm from our infusion site, a distance that is too far away to be to be directly affected by our infusions. The LH surge depends at least partly on the preoptic area and can be stimulated by noradrenergic inputs [54, 60, 66, 67] so it could have been affected by our treatment. In any case, it is difficult in ewes with “precocious LH surges” to dissociate increases in LH pulsatility and onset of the LH surge as can be done in the cyclic ewe at the end of the follicular phase [46, 68]. The mechanism involved in the switch between the pulsatile and surge mode of secretion is still not clear [60]. It could depend on different populations of GnRH neurons but this has never been clearly demonstrated or on the relative contribution of other neurotransmitters and neuropeptides [60, 69] that are modulated by many different factors: steroids, nutrition, stress, photoperiod and the circadian clock to name but a few This would allow very broad control of reproduction by environmental factors. We suggest that the “ram effect” stimulates a pathway that allows the mating partner to directly influence the secretion of GnRH and that this involves noradrenaline and has similarities with the pathways involved in the secretion of GnRH for spontaneous ovulation. In most ewes as in other “spontaneous” ovulators, the influence of this pathway is modulatory because the LH surge is driven by increasing concentration of ovarian oestradiol [70]. But in some ewes, because of a higher noradrenergic tone or perhaps a higher sensitivity to noradrenaline, the ram-induced pathway is prematurely activated in ewes with “precocious” LH surges.

The existence of neural circuits underlying the male-induced LH surge in spontaneously ovulating species was first proposed almost 40 years ago [35, 71]. The most common examples cited to support this idea are the induction of an LH surge by mating in rats exposed to constant light [72, 73] and in hypogonadal mice grafted with GnRH neurons that only have a LH surge after mating [74]. But these are quite extreme experimental perturbations. However, the male-induced LH surges were also observed in more physiological situations in a study using aged rats [75]. These females were unresponsive to oestradiol positive feedback because of persistent oestrus but had LH surges following caging with a male rat and interestingly this occurred even if intromission was not possible [75]. These studies show that the neural circuitry required for both the oestradiol driven LH surge and the male-induced LH surge are both functional in females and can be activated under appropriate conditions. The activation of this neuronal system could also be responsible for the ability of a sexual partner to advance the time of the LH surge as has been reported for the sow [76], cow [77] and ewe [78] and even if the females have been ovariectomized and treated with exogenous oestradiol [38]. Some authors have suggested that induced ovulation is the ancestral mode of preovulatory LH secretion [18, 34]. The presence of the neural circuits underlying the male-induced LH surge in aged rats or in some anoestrus ewes could be the evolutionary traces of an ancestral mode of LH secretion. Our speculation is that because the network for oestradiol positive feedback is dominant, the involvement of an ancestral circuit is restricted to conditions where the network of oestradiol positive feedback is not functional for example, in the aged rat, the hypogonadal mouse, and ovariectomized ewes [38] or as in our experiment, in anoestrous ewes with a high degree of responsiveness to a sexual partner.

Noradrenaline is involved in both the neural circuits for spontaneous and induced ovulation. The questions now are why one circuit is activated in preference to the other and how are these two circuits inter-connected.
